# Contemporary indications and outcomes of open surgical cerebral aneurysm management in the endovascular era

**DOI:** 10.3389/fsurg.2026.1811416

**Published:** 2026-06-24

**Authors:** Jonathan Rychen, Christian Ferreira, Marcio Y. Ferreira, Zoey Croft, Valentin F. Weiger, Griffin Thomas, Christian Rajkovic, Katherine Stark, Yafell Serulle, Jason A. Ellis, David J. Langer

**Affiliations:** 1 Department of Neurosurgery, Lenox Hill Hospital, Zucker School of Medicine at Hofstra/Northwell, Northwell Health, New York, NY, United States; 2 Department of Neurosurgery, University Hospital of Basel, University of Basel, Basel, Switzerland

**Keywords:** bypass, cerebral aneurysm, cerebrovascular surgery, clipping, microsurgery, open surgery, trapping

## Abstract

**Background and objective:**

In the current endovascular era, only a minority of intracranial aneurysms (IA) are treated surgically. This study aims to delineate the contemporary indications and outcomes of open microsurgical treatment.

**Methods:**

Retrospective single-center analysis of patients treated for IA between 2019 and 2023, including ruptured and unruptured cases. Our center follows an “interventional-first” strategy, reserving surgery for aneurysms deemed unsuitable for endovascular therapy. For each surgical case, the reason for endovascular unsuitability and the corresponding outcome were assessed.

**Results:**

A total of 432 aneurysms were analyzed, of which 39.1% were treated with open surgery and 60.9% with endovascular therapies. In the surgical cohort (140 patients, 169 treated aneurysms), 41.4% of cases presented with subarachnoid hemorrhage. The main morphological reasons for selecting open surgery were wide-necked aneurysms (27%), branch incorporation at the neck (19%), and very small aneurysm size (<3 mm) (8.6%). A substantial proportion of bystander aneurysms (17.2%) were treated surgically because the index aneurysm required open surgery, allowing simultaneous treatment. Prior endovascular treatment had been performed in 15.7% of surgical cases. Adjunctive techniques to clipping, including anterior or posterior clinoidectomy, were necessary in 9.3% of cases. Cerebral bypass and trapping were required in 5.7% of cases, typically for large, calcified aneurysms with stenotic parent arteries. Complete aneurysm occlusion was achieved in 93.3% of cases. Major and minor complication rates were 3.6% and 7.9%, respectively.

**Conclusion:**

This study outlines contemporary indications for open surgery in the endovascular era and supports the complementary role of microsurgery within an integrated treatment strategy. Microsurgical treatment achieved high rates of complete aneurysm occlusion with acceptable morbidity, even in complex and previously endovascularly treated lesions. Careful patient selection and the use of adjunctive skull base and bypass techniques remain essential.

## Introduction

Over the past two decades, there has been a marked shift from open surgery toward endovascular treatment for intracranial aneurysms (IA), such that the vast majority are nowadays managed using endovascular techniques (1–3). This shift highlights how aneurysm management has evolved in today's endovascular era. Numerous comparative studies and meta-analyses have evaluated clipping vs. endovascular therapy. It is well established that endovascular treatments generally achieve lower complete-occlusion rates, higher recurrence and retreatment rates, but are associated with fewer periprocedural complications and typically better functional outcomes (4–12). As technological advancements in endovascular therapies broaden their indications, the key questions become: which aneurysms still warrant or require open microsurgical treatment today? What are the contemporary indications, necessary surgical techniques, and expected outcomes? This study aims to address these questions by presenting the Lenox Hill experience.

## Methods

### Study design and inclusion criteria

This study is a retrospective analysis of a single-center cohort. All consecutive adult patients who underwent open surgical or endovascular treatment for IA at Lenox Hill Hospital (New York, NY, USA) between 2019 and 2023 were included. Both unruptured and ruptured aneurysm cases were analyzed.

### Decision making between endovascular and open surgical management

In general, each case is reviewed during our weekly interdisciplinary neurovascular conference. In emergency situations, the indication for endovascular vs. open surgical management is discussed among the neurovascular physicians on call. Our center follows a “interventional-first” strategy, meaning that endovascular treatment is generally preferred when appropriate for the patient's condition and aneurysm morphology. In cases of equipoise between both treatment options, endovascular therapy is typically favored, however, our center has excellent expertise in both endovascular and open surgical treatments.

### Outcome analysis

The variables analyzed included age, sex, aneurysm rupture status, prior treatment, aneurysm type, location, and size. Aneurysms were classified by size according to the Japanese unruptured cerebral aneurysm (UCAS) study: small (<5 mm), medium (5–10 mm), large (10.1–25 mm), and giant (>25 mm) (13, 14). Additionally, aneurysms smaller than 3 mm were defined as “very small”. A wide-neck aneurysm was defined as a neck diameter ≥4 mm or a dome-to-neck ratio <2 (15). For each surgically treated aneurysm, the rationale for deeming endovascular management unsuitable or suboptimal was assessed. Primary outcomes were aneurysm occlusion rate and procedural complications. To assess aneurysm occlusion after clipping, an intraoperative angiography was routinely performed; if not available, a postoperative angiography, CTA or MRA was obtained instead. In cases of aneurysm remnants following surgical clipping, they were classified according to the Sindou classification into categories 1–2 and 3–5 (16). Aneurysm recurrence was defined as regrowth of an aneurysm that had been previously completely occluded, whereas remnant enlargement referred to the growth of a known, partially occluded aneurysm. Complications directly related to surgery or endovascular treatment were assessed, while unrelated medical complications were excluded from the analysis. Complications were classified as either minor or major: minor complications were defined as non–life-threatening, transient, and without impact on quality of life; major complications were defined as permanent deficits affecting quality of life. The modified Rankin Scale (mRS) was used as a standardized outcome measure and was assessed preoperatively and at the latest postoperative follow-up. Any postoperative decline in mRS was considered a major complication. While the primary focus of this study was on the open surgical cohort, a concise, unmatched descriptive analysis of the endovascular cohort was also performed to outline indications and outcomes for each treatment modality. In the endovascular group, occlusion rates were assessed at the latest available follow-up.

### Statistical analysis

Descriptive statistics were conducted to summarize dataset characteristics. Categorical variables are reported as frequencies and percentages, and continuous variables as mean and standard deviation (SD) or median and interquartile range (IQR), as appropriate. Unadjusted comparisons were made between ruptured and unruptured cases and between surgical and endovascular groups. Predictors of aneurysm occlusion and complications were evaluated using Generalized Linear Models and Generalized Linear Mixed Models, with patient-level random effects. Results are reported as odds ratios with 95% confidence intervals (CI), with significance set at *p* < 0.05. Analyses were performed in R (R Core Team).

### Ethics

The study was approved by the Institutional Review Board (IRB #25-0248). Patient informed consent was obtained for all surgeries.

## Results

A total of 432 aneurysms were analyzed, of which 169 (39.1%) were treated with open surgery and 263 (60.9%) with endovascular therapies. The following sections focus on the open surgical cohort, while the final paragraph offers a descriptive summary of the surgical and endovascular cohorts.

### Baseline characteristics of the open surgical cohort

**Figure 1:**
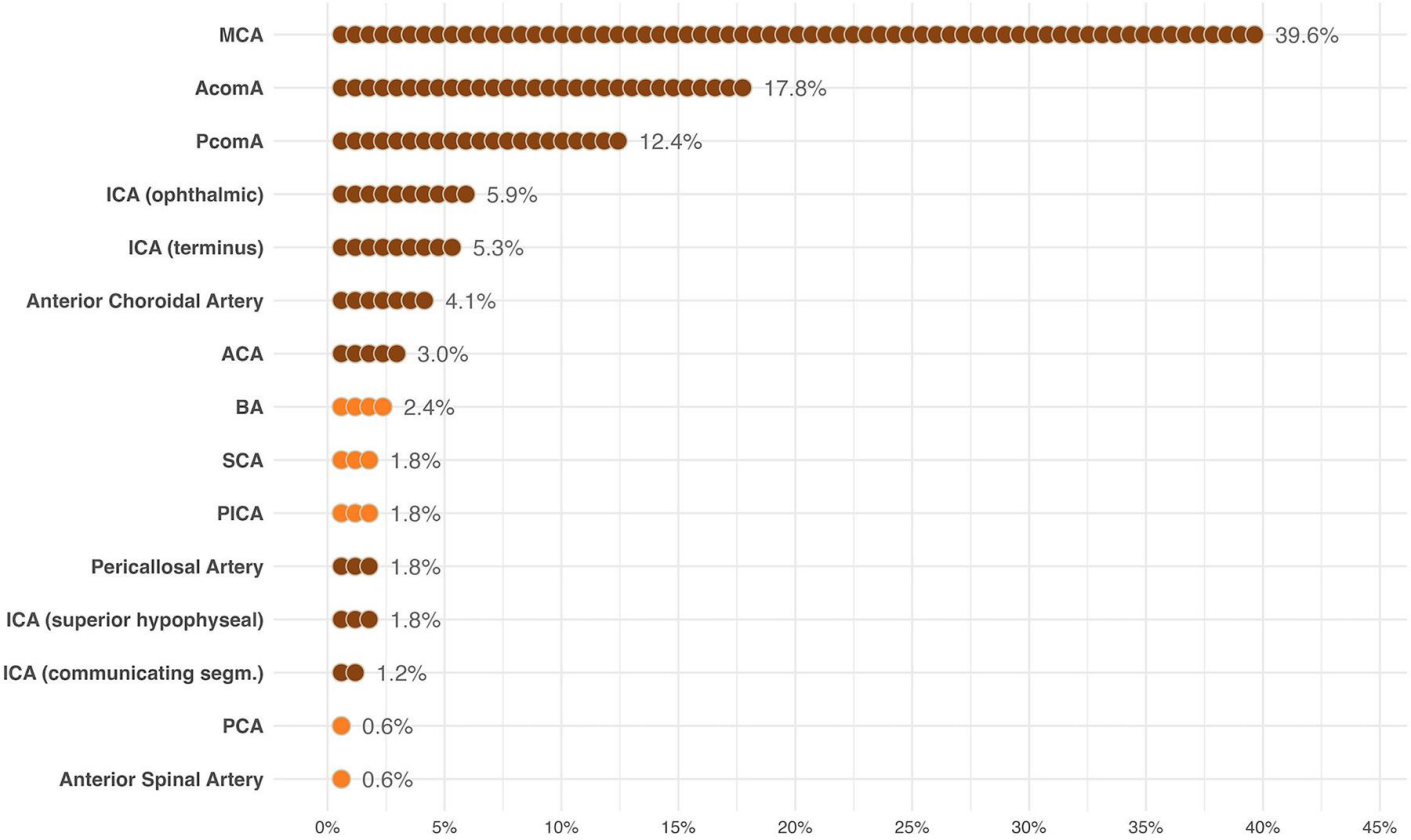
Dot-density plot illustrating the anatomical distribution of surgically treated aneurysms. Each dot represents one aneurysm. Blue dots indicate aneurysms in the anterior circulation, while red dots represent those in the posterior circulation. MCA, middle cerebral artery; AcomA, anterior communicating artery; PcomA, posterior communicating artery; ICA, internal carotid artery; ACA, anterior cerebral artery; BA, basilar artery; SCA, superior cerebellar artery; PICA, posterior inferior cerebellar artery; Segm, segment; PCA, posterior cerebral artery.

**Figure 2:**
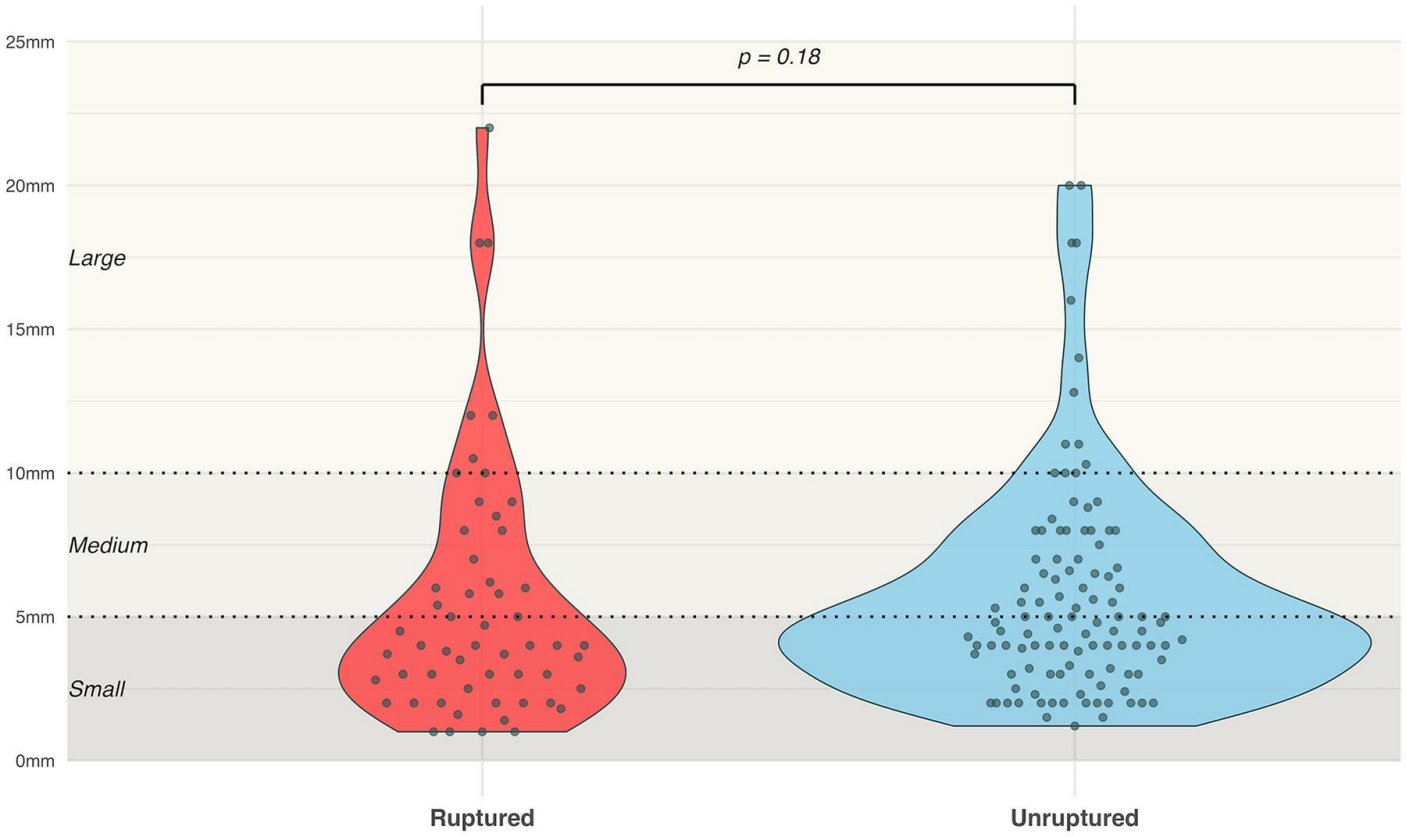
Violin plot illustrating the size distribution of surgically treated aneurysms. The red area represents the proportion of ruptured aneurysms within each size range, while the blue area represents unruptured aneurysms. Aneurysm sizes are categorized as follows: small (< 5 mm), medium (5–10 mm), and large (> 10.1 mm). The mean size of ruptured aneurysms did not differ significantly from that of the unruptured cohort.

The baseline characteristics of the open surgical cohort are presented in Table 1. A total of 169 aneurysms were treated surgically in 140 patients. Of these, 82 (58.6%) had unruptured aneurysms, while 58 (41.4%) presented with subarachnoid hemorrhage (SAH). As expected, saccular aneurysms were the most common type (*n* = 152, 94%). They were significantly more prevalent in the unruptured group compared to the ruptured group (98% vs. 89%, *p* = 0.01). Blister aneurysms were observed exclusively in the ruptured group (6.0 vs. 0%, *p* = 0.02). Figure 1 illustrates the distribution of aneurysm locations. The majority of surgically treated aneurysms were located in the anterior circulation (*n* = 157, 92.8%), with the middle cerebral artery being the most common site (*n* = 67, 39.6%). Although the mean aneurysm size was 5.6 mm (range: 1–22 mm) with no significant difference between the unruptured and ruptured groups (Table 1), Figure 2 highlights that the majority of surgically treated aneurysms were actually small (<5 mm) in both groups.

**Table 1:** Baseline characteristics of the open surgical cohort.

Variable	Entire surgical cohort (140 patients, 169 aneurysms)	Unruptured cases (82 patients, 58.6%)	Ruptured cases (58 patients, 41.4%)	*P*-value^a^
Age (years), mean ± SD	53.1 ± 13	54 ± 12.8	51.8 ± 13.4	0.32
Sex (female), *n* (%)	102 (72.9%)	62 (75.6%)	40 (69%)	0.5
Modified Rankin scale (preop), median (IQR)	1 (1–2)	1 (0–1.5)	2 (2–3)	< 0.01
SAH grading scores, median (IQR)				
Hunt and Hess scale			2 (2–3)	
Modified Fisher scale			4 (3–4)	
Aneurysm type, *n* (%)				
Saccular	152 (94.0%)	96 (98.0%)	56 (89.0%)	0.01
Blister	4 (2.0%)	0 (0%)	4 (6.0%)	0.02
Fusiform	4 (2.0%)	1 (1.0%)	3 (5.0%)	0.3
Dissecting	1 (1.0%)	1 (1.0%)	0 (0%)	1
Mycotic	0 (0%)	0 (0%)	0 (0%)	1
Aneurysm size (mm), mean (range)	5.6 (1–22)	5.7 (1.2–20)	5.4 (1–22)	0.18
Number of aneurysms by size category, *n* (%)				
Small size (< 5mm)	90 (55.2%)	57 (53.3%)	33 (58.9%)	
Medium size (5–10mm)	57 (35%)	41 (38.3%)	16 (28.6%)	
Large size (10.1–25mm)	16 (9.8%)	9 (8.4%)	7 (12.5%)	
Giant size (> 25mm)	0 (0%)	0 (0%)	0 (0%)	
Number of aneurysms treated per patient, mean (range)	1.2 (1–3)	1.2 (1–3)	1.2 (1–3)	0.83

SD, standard deviation; SAH, subarachnoid hemorrhage; IQR, interquartile range. .

a A Chi-square or Fisher’s exact test (depending on the sample size) test was used for the comparison of categorical and a Student’s t-test for continuous variables. A Mann–Whitney U test was used to compare two groups of non-normal distributed continuous or ordinal variables.

### Previously treated aneurysms in the open surgical cohort

Among the 140 cases treated surgically, 24 (17.1%) had been previously treated. Of these, 20 (91.7%) had undergone prior endovascular treatment and only 2 (8.3%) had been previously treated surgically (*p* < 0.01) (Table 2).

**Table 2:** Previously treated aneurysms in the open surgical cohort.

Variable	Previously treated surgically (n, %)	Previously treated endovascularly (n, %)	*P*-value^a^
Previously treated aneurysms (Total: *n*=24, 17.1%)	2 (8.3%)	22 (91.7%)	< 0.01
Failure of previous treatment	0 (0%)	12 (54.5%)	
Remnant or enlargement of remnant after previous treatment	2 (100%)	3 (13.6%)	
Recurrence after previous treatment	0 (0%)	7 (31.8%)	

a A Fisher’s exact test was used

### Indication for open surgical treatment

**Figure 3:**
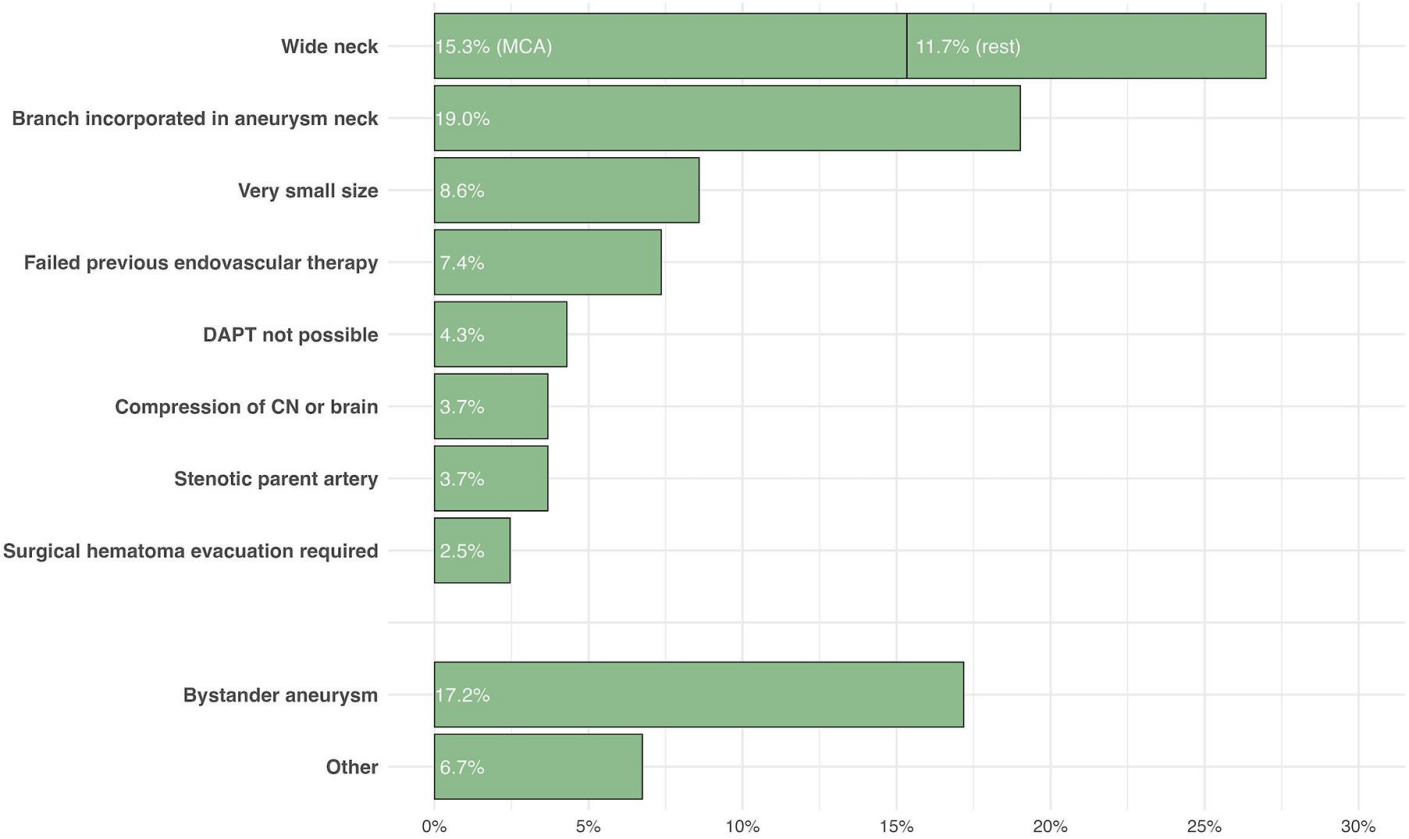
Bar plot presenting the main reasons why endovascular treatment was considered unsuitable or suboptimal for each aneurysm. MCA, middle cerebral artery; DAPT, dual antiplatelet therapy; CN, cranial nerve.

Figure 3 presents the main reasons why endovascular treatment was deemed unsuitable or suboptimal for each aneurysm. The most frequent morphological feature prompting open surgical treatment, regardless of rupture status, was a wide aneurysm neck (*n* = 44, 27%). The second most common reason was the incorporation of a vessel branch into the aneurysm neck (*n* = 31, 19%). Very small aneurysm size (< 3 mm) was a reason to favor open surgery in 8.6% (*n* = 14) of cases, due to the known increased risk of re-rupture during coiling of those small aneurysms. A prior failed endovascular treatment attempt led to the indication for surgery in 7.4% (*n* = 12) of cases. Some aneurysms were technically suitable for stent-assisted coiling or flow diverter placement, but open surgery was preferred in 4.3% (*n* = 7) of cases to avoid dual antiplatelet therapy (DAPT), in the context of SAH or other contraindications. Neurovascular decompression through open surgery was favored in 3.7% (*n* = 6) of cases where aneurysm-related compression of a cranial nerve or adjacent brain structures was present and caused neurologic symptoms or signs. A stenotic parent artery, which can complicate endovascular access or stent placement, warranted surgical treatment in another 3.7% (*n* = 6). In 2.5% (*n* = 4) of cases, surgical clipping was performed in conjunction with hematoma evacuation or decompressive craniectomy, allowing for simultaneous treatment of the ruptured aneurysm. A significant proportion of so-called *bystander* aneurysms (*n* = 28, 17.2%) were treated with open surgery simply because the *index* aneurysm required surgical management, allowing the bystander aneurysms to be addressed through the same approach.

### Adjunctive techniques to surgical clipping

An anterior or posterior clinoidectomy was performed in 13 (9.3%) cases. An anterior clinoidectomy is typically required for large internal carotid artery (ICA) aneurysms or very proximal aneurysms, such as clinoidal or ophthalmic segment aneurysms. Posterior clinoidectomy is generally indicated for low-riding basilar artery (BA) and superior cerebellar artery (SCA) aneurysms.

A cerebral bypass procedure was required in 8 (5.7%) cases. Indications and outcomes are presented in Table 3. The typical indication for bypass and aneurysm trapping included large, dysplastic, calcified, and/or thrombosed aneurysms with a stenotic parent artery and/or incorporated branches. Extracranial-intracranial (EC-IC) bypass was the most commonly performed revascularization type in our series. Intraoperative bypass patency was confirmed in all cases; however, two anastomoses occluded in the early postoperative period, resulting in ischemic strokes. All aneurysms treated with bypass and trapping were completely occluded with no evidence of recurrence at the mean follow-up (FU) of 2.1 years.

**Table 3:** Indication, type and outcome of cerebral bypass for complex aneurysm treatment.

Case	Indication	Craniotomy/Approach	Bypass Type	Bypass Name	Bypass Patency (at last FU)	Aneurysm Obliteration/Recurrence (at last FU)	Complication
1	L unrup. dysplastic MCA-bif. aneurysm (11mm) with incorporated M2	Pterional/Transsylvian	Combination (In-situ + EC-IC)	M2-M2 *in situ* and STA-M2; Aneurysm trapping	Patent (at 5 yrs)	Complete/No recurrence (at 5 yrs)	Minor (transient deficit)
2	L unrup. MCA-bif. aneurysm (10mm) with stenotic parent artery and ischemia	Pterional/Transsylvian	EC-IC	Double barrel STA-M2; Aneurysm trapping	Patent (at 8.6 mo)	Complete/No recurrence (at 8.6 mo)	Minor (seizure)
3	L previously rupt. M2 fusiform aneurysm (16mm)	Orbitozygomatic/Transsylvian	EC-IC interpositional	Imax-ATib-M2; Aneurysm trapping	Patent (at 6 yrs)	Complete/No recurrence (at 6 yrs)	None
4	R unrupt. dysplastic CmaA aneurysm (8mm) with stenotic parent artery	Orbito-frontotemporal/Interhemispheric	EC-IC	STA-CmaA; Aneurysm trapping	Patent (at 6 mo)	Complete/No recurrence (at 6 mo)	Minor (seizure)
5	R rupt. M1 fusiform and dysplastic aneurysm (12mm)	Frontotemporal/Transsylvian	EC-IC	STA-M2; Aneurysm trapping	Initially patent, occluded at POD 1	Complete/No recurrence (at 9 mo)	Major (ischemic stroke)
6	L unrupt. calcified and partially thrombosed ICA (ophthalmic) aneurysm (15mm) with stenotic parent artery	Pterional/Transsylvian and subfrontal	EC-IC	STA-M2; Aneurysm trapping	Patent (at 10 mo)	Complete/No recurrence (at 10 mo)	None
7	R rupt. fusiform PICA aneurysm (3.5mm)	Median suboccipital	EC-IC	OA-PICA; Coil occlusion of aneurysm	Patent (lost to FU after discharge)	Complete (lost to FU after discharge)	None
8	R unrupt. dissecting and partially thrombosed P2 aneurysm (8mm) with stenotic parent artery	Frontotemporal/Transsylvian and subtemporal	EC-IC	STA-P2; Aneurysm trapping	Initially patent, occluded at POD 5	Complete/No recurrence (at 3 yrs)	Major (ischemic stroke)

L, left; R, right; Unrup, unruptured; Rupt, ruptured; MCA, middle cerebral artery; Bif, bifurcation; EC, extracranial; IC, intracranial; STA, superficial temporal artery; Yrs, years; FU, follow-up; Mo, month; Imax, internal maxillary artery; ATib, anterior tibial artery; CmaA, callosomarginal artery; POD, postoperative day; PICA, posterior inferior cerebellar artery; OA, occipital artery .

### Outcomes after surgical aneurysm treatment

**Figure 4:**
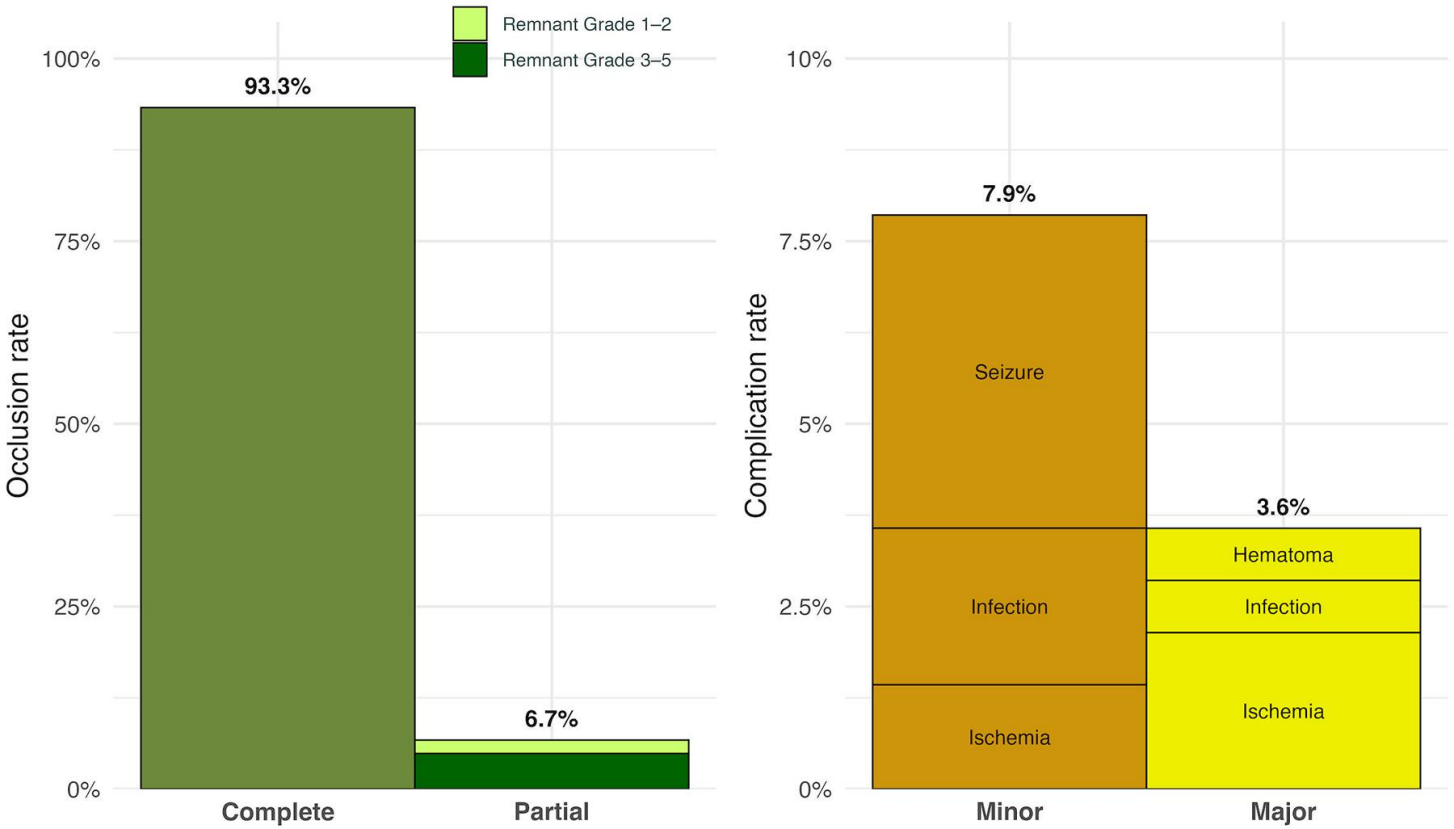
Bar plot illustrating the efficacy and safety profile of open surgery. The left panel shows aneurysm occlusion rates, with remnants classified according to the system by Sindou et al. (16). The right panel displays complication rates, categorized as either minor or major. Minor complications were defined as non–life-threatening, transient, and without impact on quality of life; major complications were defined as permanent deficits that affect quality of life.

The primary outcomes, including aneurysm occlusion and complication rates, are presented in Figure 4. The aneurysm occlusion rate was determined using the following imaging modalities: intraoperative angiography in 117 (83.6%) cases, postoperative angiography only in 11 (7.9%) cases, postoperative CTA only in 8 (5.7%) cases, and postoperative MRA only in 1 (0.7%) case; imaging was unavailable in 3 (2.1%) cases. Based on these imaging modalities, the rate of complete aneurysm occlusion was 93.3% (*n* = 153). Among the 11 aneurysms (6.7%) with partial occlusion, 3 (27.3%) were classified as remnant grade 1–2, and 8 (72.7%) as remnant grade 3–5 on intraoperative or postoperative angiography. Although no predictor reached the conventional threshold for statistical significance (*p* < 0.05), aneurysm size demonstrated a trend toward significance, suggesting that larger aneurysms may be associated with reduced odds of complete occlusion (OR 0.89; 95% CI 0.79–1.01; *p* = 0.054). Similarly, ruptured aneurysms showed a borderline association with lower occlusion rates (OR 0.31; 95% CI 0.08–1.10; *p* = 0.076). During the short median follow-up of 112 days (IQR: 2–334), there were no recurrences of completely occluded aneurysms, no remnant growth, and no cases of bleeding or rebleeding from treated aneurysms. However, 5 patients (3.6%) required retreatment of a partially clipped aneurysm, all of which were managed using an endovascular technique.

Regarding complications, there were no intraoperative or in-hospital deaths in this surgical cohort. The rate of major complications was 3.6% (*n* = 5), including ischemic strokes (*n* = 3), a postoperative infection (*n* = 1), and a hematoma (*n* = 1). Minor complications occurred in 7.9% of cases (*n* = 11), consisting of postoperative seizures (*n* = 6), infections (*n* = 3), and ischemic strokes (*n* = 2). No predictors of complications could be identified in this cohort due to the low number of events, which limited the statistical power needed for such analysis.

### Comparative summary of surgical and endovascular cohorts

On average, patients in the endovascular group were older than those in the clipping group. The endovascular cohort was more likely to have a single treated aneurysm, while multiple aneurysms were more common in the clipping group. Regarding aneurysm location, clipping was more frequent for middle cerebral aneurysm (MCA) aneurysms, whereas endovascular treatment was more commonly used for ICA aneurysms. In terms of outcomes, the clipping cohort had a higher rate of complete aneurysm occlusion compared to the endovascular cohort. Conversely, the endovascular group showed higher rates of retreatment. Complication rates were comparable between the groups. The median hospital stay, regardless of rupture status, was shorter in the endovascular cohort (Table 4).

**Table 4:** Unmatched comparison of the open surgical with the endovascular cohort.

Variable	Open surgical cohort (140 patients, 169 aneurysms)	Endovascular cohort (254 patients, 263 aneurysms)	*P* value^a^
Baseline characteristics			
Age (years), mean ± SD	53.1 ± 13	57.4 ± 14.3	0.004
Sex (female), *n* (%)	102 (72.9%)	176 (72.7%)	1
Ruptured, *n* (%)	58 (41.4%)	103 (39.0%)	0.976
SAH grading scores, median (IQR)			
Hunt and Hess Grade	2 (2–3)	3 (2–4)	0.327
Modified Fisher Grade	4 (3–4)	3 (3–4)	0.315
Number of aneurysms treated, *n* (%)			
1	113 (67.0%)	224 (85.0%)	<0.001
2	17 (10.0%)	14 (5.0%)	0.018
3	6 (4.0%)	4 (2.0%)	0.993
Aneurysm location, *n* (%)			
ACA	5 (3.0%)	5 (2.0%)	0.695
ACOM	30 (18.0%)	57 (22.0%)	0.395
Ant. choroidal	7 (4.0%)	4 (2.0%)	0.167
Anterior spinal	1 (1.0%)	1 (0.0%)	1
BA	4 (2.0%)	17 (6.0%)	0.09
ICA cavernous segment	0 (0.0%)	5 (2.0%)	<0.001
ICA communicating segment	2 (1.0%)	15 (6.0%)	0.036
ICA ophthalmic segment	10 (6.0%)	49 (19.0%)	<0.001
ICA superior hypophyseal	3 (2.0%)	23 (9.0%)	0.006
ICA terminus	9 (5.0%)	7 (3.0%)	0.239
MCA	67 (40.0%)	12 (5.0%)	<0.001
PCA	1 (1.0%)	3 (1.0%)	0.95
PCOM	21 (12.0%)	39 (15.0%)	0.584
Pericallosal	3 (2.0%)	9 (3.0%)	0.477
PICA	3 (2.0%)	6 (2.0%)	0.993
SCA	3 (2.0%)	4 (2.0%)	1
Aneurysm type, *n* (%)	161 (95.0%)	260 (99.0%)	-
Saccular	152 (94.0%)	236 (90.0%)	0.984
Fusiform	4 (2.0%)	19 (7.0%)	0.049
Blister	4 (2.0%)	2 (1.0%)	0.329
Dissecting	1 (1.0%)	3 (1.0%)	0.95
Mycotic	0 (0%)	0 (0%)	1
Aneurysm size category, *n* (%)			
Small (<5mm)	90 (53.0%)	113 (43.0%)	0.043
Medium (5–10mm)	57 (34.0%)	116 (44.0%)	0.044
Large (10.1–25mm)	16 (9.0%)	33 (13.0%)	0.414
Giant (>25mm)	0 (0%)	0 (0%)	1
Previously treated aneurysms	24 (17.1%)	18 (6.9%)	0.045
Outcomes			
Complete aneurysm occlusion, *n* (%)	155 (93.3%)	180 (68.7%)	<0.001
Recurrence, *n* (%)	0 (0.0%)	37 (14.1%)	<0.001
Need for re-treatment, *n* (%)	5 (3.0%)	45 (17.2%)	<0.001
Rebleeding of a treated aneurysm, *n* (%)	0 (0.0%)	0 (0.0%)	NA
Complications (overall), *n* (%)	15 (11.5%)	35 (13.3%)	0.47
Major complication, *n* (%)	5 (3.6%)	9 (3.4%)	1
Minor complications, *n* (%)	10 (7.9%)	26 (9.9%)	0.205
Intraop death, *n* (%)	0 (0.0%)	0 (0.0%)	NA
In hospital death, *n* (%)	0 (0.0%)	8 (3.0%)	<0.001
Length of stay (days), median (IQR)	8.5 (3–20)	2 (1–16)	<0.001

SAH, subarachnoid hemorrhage; IQR, interquartile range; ACA, anterior cerebral artery; ACOM, anterior communicating artery; MCA, middle cerebral artery; Ant, anterior; BA, basilar artery; ICA, internal carotid artery; PCA, posterior cerebral artery; PCOM, posterior communicating artery; PICA, posterior inferior cerebellar artery; SCA, superior cerebellar artery; NA, non applicable.

a The Mann–Whitney U test was used for comparing continuous variables, and a proportion test with continuity correction was used for comparison of categorical variables. .

### Illustrative cases

#### Case 1

Example of a large 14 mm ICA aneurysm with stenotic parent artery requiring superficial temporal artery (STA) to MCA bypass and aneurysm trapping (Supplementary Video 1).

#### Case 2

Example of an anterior clinoidectomy and clipping of a residual posterior communicating artery (PCOM) aneurysm after failure of endovascular treatment. The patient's neck was prepped in advance to permit proximal control of the cervical carotid artery if required (Supplementary Video 2).

#### Case 3)

Example of a very small ruptured posterior cerebral artery (PCA) perforator aneurysm treated with surgical clipping (Supplementary Video 2).

## Discussion

This study offers timely insight into the role of open surgery in the modern endovascular era. Moreover, it illustrates the value of a multidisciplinary team approach combined with an “interventional-first” treatment philosophy for IA. Endovascular therapy was prioritized when feasible, while microsurgical clipping was reserved for cases considered suboptimal or unsuitable for catheter-based treatment. Within this framework, we observed good surgical outcomes. By clearly outlining the morphological and clinical reasons for selecting surgery, this study helps define the niche in which microsurgery remains essential. Although many publications compare the outcomes of clipping vs. coiling, very few provide a detailed comparative analysis of indications within a dual-modality, single-center program (17–20). This highlights the relative scarcity of studies structured similarly to ours. Randomized trials are unlikely to resolve the question of the best treatment modality, as true equipoise rarely exists. A team-based, individualized decision-making process should be applied to every patient. The slightly better surgical outcomes in our cohort should not be viewed as evidence that microsurgery is inherently safer than endovascular therapy. Rather, they reflect case selection and emphasize the importance of maintaining high-level microsurgical expertise. Centers that rely almost exclusively on catheter-based strategies may inadvertently limit optimal treatment options and risk poorer outcomes; an increasingly relevant concern given the shrinking availability of open cerebrovascular surgery in the United States. The central message is not that surgery is superior to endovascular therapy. The central message is that centers treating aneurysms must maintain both options at a high level, as the absence of a robust open surgical alternative may compromise overall group outcomes.

It is not surprising that the MCA was the most common site treated with open surgery, as this aligns with the literature due to its favorable surgical accessibility, and the frequent occurrence of wide-necked aneurysms, often with incorporated branches (12, 21). What is more surprising in this cohort is the size of the ruptured aneurysms. The majority measured less than 5 mm, a finding of interest as it falls below the average rupture size commonly reported in the literature, which is approximately 7 mm (22). However, some studies have shown that the size of ruptured aneurysms has decreased over the years, although the underlying reasons for this trend remain unclear (23). The fact that the majority of ruptured aneurysms measured less than 5 mm justifies why so many small unruptured aneurysms were treated in our cohort. However, in the unruptured cohort, a bias arises from the presence of bystander aneurysms. The decision for open surgery was based on the index aneurysm, which is typically larger than the accompanying bystanders. Notably, 17.2% of clipped aneurysms were bystanders, thereby skewing the overall size distribution of treated unruptured aneurysms toward smaller sizes.

This study reinforces the role of open surgery in managing aneurysms with complex morphological features or when endovascular options are limited. The most frequent reason for choosing surgery was a wide aneurysm neck, which is known to increase the risk of coil instability and incomplete occlusion (24). Stent-assisted coiling is a suitable alternative for unruptured wide-necked aneurysms; however, some patients either decline or have medical contraindications to DAPT, as observed in our series. Newer techniques, such as the Woven EndoBridge (WEB) device, have not demonstrated sufficiently high occlusion rates to justify replacing surgical treatment for those aneurysms (25). Vessel branches incorporated into the aneurysm neck also commonly led to surgical management, as clipping allows for better preservation of critical vessels to avoid ischemic complications (26). Very small aneurysms were another indication, given the higher risk of re-rupture during endovascular manipulation (27). Regarding previously treated aneurysms, our findings suggest that those requiring surgical retreatment were predominantly cases of failed prior endovascular management. This reinforces the continued importance of open surgery as a definitive treatment option, serving in many cases as a form of “salvage therapy” (28–31).

The high complete occlusion rates achieved with surgical clipping are consistent with prior reports (4–12). Together with low recurrence and retreatment rates, these findings support surgery, particularly in younger patients with longer life expectancy. Notably, despite the increasing complexity of aneurysms treated today (32), clipping still achieved high complete occlusion with an acceptable complication profile and no mortality in our cohort. This study also underscores the need for neurovascular surgeons to master microsurgical techniques beyond standard clipping (28). A significant proportion of complex aneurysms required skull base approaches, including anterior and/or posterior clinoidectomy and cerebral bypass, to achieve the best possible outcomes.

The challenge with selecting surgery is that it remains an “open” procedure. The threshold not only for physicians to recommend, but also for patients to accept open surgery continues to rise. Early outcomes are clearly more favorable with catheter-based treatments, particularly when considering shorter hospital stays and the absence of a surgical scar and postoperative pain. For this reason, when appropriate and safe, minimally invasive surgical approaches such as the minipterional or supraorbital craniotomy should be increasingly utilized, to optimize patient outcomes and align with modern surgical standards (33–37). Additionally, ongoing refinement of these keyhole techniques is essential to further minimize the surgical footprint and remain competitive with evolving endovascular therapies (38).

We would like to emphasize that continuous neurosurgical training remains essential for the open cerebrovascular treatment of aneurysms that are not amenable to endovascular therapy, as well as for cases in which endovascular treatment fails or results in acute complications. Due to the increasing use of endovascular techniques, cerebrovascular surgeons are now confronted with fewer cases overall, but a greater proportion of highly complex aneurysms. Management of these lesions may require technically demanding bypass or skull base techniques, underscoring the need for sustained technical mastery (28). Such expertise should be an integral component of any center treating cerebral aneurysms; otherwise, these cases should only be managed by teams capable of providing the necessary microsurgical expertise. The importance of vascular microneurosurgical training for the next generation of neurosurgeons cannot be overstated. Developing and maintaining expertise in complex aneurysm surgery requires lifelong dedication and continuous training. Exposure during residency and fellowship is often insufficient; therefore, young cerebrovascular surgeons should actively pursue ongoing additional training through cadaveric dissections, microsurgical laboratory practice, and courses at high-volume specialized centers to acquire and refine these advanced surgical skills (39).

### Limitations

The main limitation of this study is its retrospective design. A second limitation is that the results reflect a single-center treatment strategy and may not be generalizable to other centers. The follow-up period was also too short to reliably assess aneurysm recurrence or retreatment. Comparisons between unmatched surgical and endovascular cohorts may have introduced baseline differences. Although *p*-values were used to highlight differences, these analyses do not adjust for confounding. Therefore, the results should be interpreted cautiously as associations rather than causal effects.

## Conclusion

This study outlines contemporary indications for open surgery in the endovascular era, supporting the complementary role of microsurgery within an integrated treatment strategy and emphasizing the importance of a multidisciplinary, individualized decision-making process. Patients selected for surgical treatment are typically younger, have multiple aneurysms, harbor wide-necked or morphologically complex lesions, or have experienced failure or recurrence following endovascular therapy. Key adjunctive surgical techniques that should be mastered include anterior and posterior clinoidectomy, as well as cerebral by bypass. Open surgery continues to achieve high rates of aneurysm occlusion with an acceptable complication profile.

## Data Availability

The raw data supporting the conclusions of this article will be made available by the authors, without undue reservation.

## Supplementary material

Supplementary Video 1 Case 1: Example of a large 14mm ICA aneurysm with stenotic parent artery requiring bypass and trapping. ED, emergency department.

Supplementary Video 2 Case 2: Example of an anterior clinoidectomy and clipping of a residual posterior communicating artery (PCOM) aneurysm after failure of endovascular treatment. The patient’s neck was prepped in advance to permit proximal control of the cervical carotid artery if required. Case 3: Example of a small ruptured posterior cerebral artery (PCA) perforator aneurysm treated with surgical clipping. CN, cranial nerve; ICA, internal carotid artery.

## References

[1] McDonald JS McDonald RJ Fan J Kallmes DF Lanzino G Cloft HJ. Comparative effectiveness of unruptured cerebral aneurysm therapies: propensity score analysis of clipping versus coiling. Stroke. (2013) 44(4):988–94. 10.1161/STROKEAHA.111.000196 23449260

[2] Bekelis K Goodney PR Dzebisashvili N Goodman DC Bronner KK. Variation in the Care of Surgical Conditions: Cerebral Aneurysms: A Dartmouth Atlas of Health Care Series. Lebanon, NH: The Dartmouth Atlas of Health Care (2014). 36454935

[3] Eskey CJ Meyers PM Nguyen TN Ansari SA Jayaraman M McDougall CG. Indications for the performance of intracranial endovascular neurointerventional procedures: a scientific statement from the American Heart Association. Circulation. (2018) 137(21):e661–89. 10.1161/CIR.0000000000000567 29674324

[4] Molyneux AJ Kerr RS Yu LM Clarke M Sneade M Yarnold JA. International subarachnoid aneurysm trial (ISAT) of neurosurgical clipping versus endovascular coiling in 2143 patients with ruptured intracranial aneurysms: a randomised comparison of effects on survival, dependency, seizures, rebleeding, subgroups, and aneurysm occlusion. Lancet. (2005) 366(9488):809–17. 10.1016/S0140-6736(05)67214-5 16139655

[5] Molyneux AJ Kerr RS Birks J Ramzi N Yarnold J Sneade M. Risk of recurrent subarachnoid haemorrhage, death, or dependence and standardised mortality ratios after clipping or coiling of an intracranial aneurysm in the international subarachnoid aneurysm trial (ISAT): long-term follow-up. Lancet Neurol. (2009) 8(5):427–33. 10.1016/S1474-4422(09)70080-8 19329361 PMC2669592

[6] Spetzler RF McDougall CG Zabramski JM Zabramski JM Albuquerque FC Hills NK. Ten-year analysis of saccular aneurysms in the barrow ruptured aneurysm trial. J Neurosurg. (2019) 132(3):771–6. 10.3171/2018.8.JNS181846 30849758

[7] Jiang Z Chen Y Zeng C Feng J Wan Y Zhang X. Neurosurgical clipping versus endovascular coiling for patients with intracranial aneurysms: a systematic review and meta-analysis. World Neurosurg. Jun. (2020) 138:e191–222. 10.1016/j.wneu.2020.02.091 32105881

[8] Smith TR Cote DJ Dasenbrock HH Hamade YJ Zammar SG El Tecle NE. Comparison of the efficacy and safety of endovascular coiling versus microsurgical clipping for unruptured middle cerebral artery aneurysms: a systematic review and meta-analysis. World Neurosurg. (2015) 84(4):942–53. 10.1016/j.wneu.2015.05.073 26093360

[9] Falk Delgado AL Andersson T Falk Delgado AN. Clinical outcome after surgical clipping or endovascular coiling for cerebral aneurysms: a pragmatic meta-analysis of randomized and non-randomized trials with short- and long-term follow-up. J Neurointerv Surg. Mar. (2017) 9(3):264–77. 10.1136/neurintsurg-2016-012292 27053705

[10] Lanzino G Murad MH D’Urso PI Rabinstein AA. Coil embolization versus clipping for ruptured intracranial aneurysms: a meta-analysis of prospective controlled published studies. AJNR Am J Neuroradiol. (2013) 34(9):1764–8. 10.3174/ajnr.A3515 23578672 PMC7965621

[11] Shao B Wang J Chen Y He X Chen H Peng Y. Clipping versus coiling for ruptured intracranial aneurysms: a meta-analysis of randomized controlled trials. World Neurosurg. (2019) 127:e353–65. 10.1016/j.wneu.2019.03.123 30928577

[12] Ferreira MY Batista S Brenner LBO Marques GN Maia HG Palavani LB. Comparing surgical clipping with endovascular treatment for unruptured middle cerebral artery aneurysms: a systematic review and updated meta-analysis. J Neurosurg. (2025) 142(1):116–26. 10.3171/2024.4.JNS24343 39094183

[13] UCAS Japan Investigators, Morita A Kirino T Hashi K Aoki N Fukuhara S. The natural course of unruptured cerebral aneurysms in a Japanese cohort. N Engl J Med. Jun. (2012) 366(26):2474–82. 10.1056/NEJMoa1113260 22738097

[14] Merritt WC Berns HF Ducruet AF Becker TA. Definitions of intracranial aneurysm size and morphology: a call for standardization. Surg Neurol Int. (2021) 12:506. 10.25259/SNI_576_2021 34754556 PMC8571384

[15] Hendricks BK Yoon JS Yaeger K. Wide-neck aneurysms: systematic review of the neurosurgical literature with a focus on definition and clinical implications. J Neurosurg. (2020) 133(1):159–65. 10.3171/2019.3.JNS183160 31200376

[16] Sindou M Acevedo JC Turjman F. Aneurysmal remnants after microsurgical clipping: classification and results from a prospective angiographic study (in a consecutive series of 305 operated intracranial aneurysms). Acta Neurochir (Wien). (1998) 140(11):1153–9. 10.1007/s007010050230 9870061

[17] Liao CC Huang YH Fang PH Lee TC. Surgical and endovascular treatment for ruptured anterior circulation cerebral aneurysms: a comparison of outcomes–a single centre study from Taiwan. Int J Surg. (2013) 11(9):998–1001. 10.1016/j.ijsu.2013.05.038 23770195

[18] Cheng-Chuan H Yu-Han H Hsuan-Yu C Chih-Wei W Chao-Bao L. Clinical outcomes of surgical and endovascular treatment of ruptured aneurysms in the anterior cerebral circulation: a single-center experience. J Radiol Sci. (2023) 48:e00004. 10.4103/jradiolsci.JRADIOLSCI-D-23-00004

[19] Sangram Bal SG Agrawal M Kumar Choudhary S Babal R Vasudeva S. Clinical outcomes of aneurysm treatment: a single-centre experience with microsurgical clipping and endovascular coiling in 1445 patients. J Cerebrovasc Sci. (2025) 13:29–34. 10.4103/jcvs.jcvs_6_25

[20] Klompenhouwer EG Dings JT van Oostenbrugge RJ Oei S Wilmink JT van Zwam WH. Single-center experience of surgical and endovascular treatment of ruptured intracranial aneurysms. AJNR Am J Neuroradiol. Mar. (2011) 32(3):570–5. 10.3174/ajnr.A2326 PMC8013111 21349958

[21] Rinne J Hernesniemi J Niskanen M Vapalahti M. Analysis of 561 patients with 690 middle cerebral artery aneurysms: anatomic and clinical features as correlated to management outcome. Neurosurgery. (Jan 1996) 38(1):2–11. 10.1097/00006123-199601000-00002 8747945

[22] Waqas M Chin F Rajabzadeh-Oghaz H Gong AD Rai HH Mokin M. Size of ruptured intracranial aneurysms: a systematic review and meta-analysis. Acta Neurochir (Wien). (2020) 162(6):1353–62. 10.1007/s00701-020-04291-z 32215742

[23] Korja M Kivisaari R Rezai Jahromi B Lehto H. Size of ruptured intracranial aneurysms is decreasing: twenty-year long consecutive series of hospitalized patients. Stroke. (2018) 49(3):746–9. 10.1161/STROKEAHA.117.019235 29371432

[24] Mascitelli JR Lawton MT Hendricks BK Nakaji P Zabramski JM Spetzler RF. Analysis of wide-neck aneurysms in the barrow ruptured aneurysm trial. Neurosurgery. (2019) 85(5):622–31. 10.1093/neuros/nyy439 30346618

[25] Fujimoto M Lylyk I Bleise C Albiña P Chudyk J Lylyk P. Long-Term outcomes of the WEB device for treatment of wide-neck bifurcation aneurysms. AJNR Am J Neuroradiol. Jun. (2020) 41(6):1031–6. 10.3174/ajnr.A6548 PMC7342766 32467180

[26] Versyck G van Loon J Lemmens R Demeestere J Bonne L Peluso JP. An overview of decision-making in cerebrovascular treatment strategies: part II—ruptured aneurysms. Brain Spine. (2024) 4:103330. 10.1016/j.bas.2024.103330 39318854 PMC11421264

[27] Mitchell PJ Muthusamy S Dowling R Yan B. Does small aneurysm size predict intraoperative rupture during coiling in ruptured and unruptured aneurysms? J Stroke Cerebrovasc Dis. (2013) 22(8):1298–303. 10.1016/j.jstrokecerebrovasdis.2012.10.017 23265780

[28] Lawton MT Lang MJ. The future of open vascular neurosurgery: perspectives on cavernous malformations, AVMs, and bypasses for complex aneurysms. J Neurosurg. (2019) 130(5):1409–25. 10.3171/2019.1.JNS182156 31042667

[29] Liu JJ Nielsen TH Abhinav K Lee J Han SS Marks MP. Surgical treatment of recurrent previously coiled and/or stent-coiled intracerebral aneurysms: a single-center experience in a series of 75 patients. World Neurosurg. Apr. (2019) 124:e649–58. 10.1016/j.wneu.2018.12.171 30639494

[30] Griessenauer CJ Dodier P Stroh NH Mercea PA Bavinzski G Dorfer C. Open microsurgical cerebral aneurysm treatment after failed endovascular therapy: an evaluation of aneurysm treatment frequencies in all neurovascular centers across Austria and the Czech republic over 20 years. Neurosurgery. (2024) 95(6):1349–57. 10.1227/neu.0000000000003040 38864626

[31] Roy AK Philipp LR Howard BM Cawley CM Grossberg JA Barrow DL. Microsurgical treatment of cerebral aneurysms after previous endovascular therapy: single-center series and systematic review. World Neurosurg. (2019) 123:e103–15. 10.1016/j.wneu.2018.11.065 30465952

[32] Mason AM Cawley CM Barrow DL. Surgical management of intracranial aneurysms in the endovascular era: review article. J Korean Neurosurg Soc. (2009) 45(3):133–42. 10.3340/jkns.2009.45.3.133 19352474 PMC2666114

[33] Zumofen DW Rychen J Roethlisberger M Taub E Kalbermatten D Nossek E. A review of the literature on the transciliary supraorbital keyhole approach. World Neurosurg. (2017) 98:614–24. 10.1016/j.wneu.2016.10.110 27989977

[34] Rychen J Croci D Roethlisberger M Nossek E Potts MB Radovanovic I. Keyhole approaches for surgical treatment of intracranial aneurysms: a short review. Neurol Res. (2018) 41:1–9. 10.1080/01616412.2018.1531202 30311865

[35] Rychen J Croci D Roethlisberger M Nossek E Potts M Radovanovic I. Minimally invasive alternative approaches to pterional craniotomy: a systematic review of the literature. World Neurosurg. (2018) 113:163–79. 10.1016/j.wneu.2018.02.016 29452317

[36] Rychen J Zumofen DW Riina HA Mariani L Guzman R. The transpalpebral versus the transciliary variant of the supraorbital keyhole approach: anatomic concepts for aneurysm surgery. Oper Neurosurg (Hagerstown). (2020) 19(1):E24–31. 10.1093/ons/opz358 31828349

[37] Rychen J Saemann A Gehweiler JE Roethlisberger M Soleman J Hutter G. The sylvian keyhole approach for surgical clipping of middle cerebral artery aneurysms: technical nuance to the minipterional craniotomy. Front Surg. (2022) 9:1078735. 10.3389/fsurg.2022.1078735 36605165 PMC9810108

[38] Davies JM Lawton MT. Advances in open microsurgery for cerebral aneurysms. Neurosurgery. Feb. (2014) 74(Suppl 1):S7–16. 10.1227/NEU.0000000000000193 24402495

[39] Burkhardt JK Lawton MT. Training young neurosurgeons in open microsurgical aneurysm treatment. World Neurosurg. (2017) 103:919–20. 10.1016/j.wneu.2017.04.089 28438650

